# Identification of bleeding points after bile duct biopsy using red dichromatic imaging during peroral cholangioscopy: A case report

**DOI:** 10.1002/deo2.215

**Published:** 2023-02-22

**Authors:** Yusuke Kimura, Naoki Okano, Kensuke Hoshi, Shuntaro Iwata, Wataru Ujita, Yuto Yamada, Susumu Iwasaki, Kensuke Takuma, Ken Ito, Yoshinori Igarashi, Takahisa Matsuda

**Affiliations:** ^1^ Division of Gastroenterology and Hepatology Toho University Omori Medical Center Tokyo Japan

**Keywords:** bile duct biopsy, bile duct bleeding, bleeding point, peroral cholangioscopy, red dichromatic imaging

## Abstract

Red dichromatic imaging is a new image‐enhancement technology that clarifies the differences in blood concentrations and improves the visibility of the bleeding point. A 71‐year‐old man was presented with a common bile duct stone, which was completely removed using electrohydraulic shock wave lithotripsy with peroral cholangioscopy. During peroral cholangioscopy, a nodular lesion was found at the confluence of the cystic duct, and a forceps biopsy was performed. It was difficult to confirm the bleeding point using white‐light imaging because of the pooling of blood. After switching to red dichromatic imaging mode 2 and washing the bile duct with saline solution, the bleeding point was observed in darker yellow than the surrounding blood, allowing the identification of the bleeding point. Red dichromatic imaging can be used in the future to maintain hemostasis during peroral cholangioscopy.

## INTRODUCTION

Red dichromatic imaging (RDI) is a new image‐enhancement technology that was released in July 2020. The RDI consists of three wavelengths: 540, 600, and 630 nm. The RDI reveals differences in blood concentrations. Most waves with a wavelength of 600 nm are unattenuated at low blood concentrations, whereas they are attenuated in the presence of high blood concentrations. Amber light with a wavelength of 600 nm is strongly attenuated at the bleeding point where the blood concentration is high; therefore, the reflectance is higher in the surrounding area where the blood concentration is low. Red light with a wavelength of 630 nm is weakly absorbed by hemoglobin, and the reflected light is hardly attenuated, regardless of the hemoglobin concentration. These absorbance differences provide color contrast and improve the visibility of the bleeding points.

Although there are few reports on the use of RDI in peroral cholangioscopy (POCS), this is the first case report in which RDI clearly identified the bleeding points after biopsy during POCS.

## CASE REPORT

A 71‐year‐old man had a history of surgery for rectal cancer and a lung metastatic tumor. The follow‐up computed tomography scan revealed a 20 mm‐sized common bile duct stone. Endoscopic retrograde cholangiopancreatography was performed at another hospital, but the stone could not be removed because it was large and hard, and the patient was referred to our hospital for treatment.

Endoscopic retrograde cholangiopancreatography was repeated at our hospital, which revealed the presence of a large stone in the lower bile duct. First, we attempted to remove the stone using a mechanical lithotripter, but the stone could not be crushed because of its hardness. Therefore, we attempted to crush the stone using electrohydraulic shock wave lithotripsy with POCS. POCS was performed using the mother–baby scope method with saline injection. The mother scope was Olympus TJF‐260V (Olympus, Tokyo, Japan), and the baby scope was CHF‐B290 (Olympus). An EVIS X1 system (Olympus) was used as the observation equipment. After electrohydraulic shock wave lithotripsy with POCS was performed, the stone was completely removed.

**FIGURE 1:**
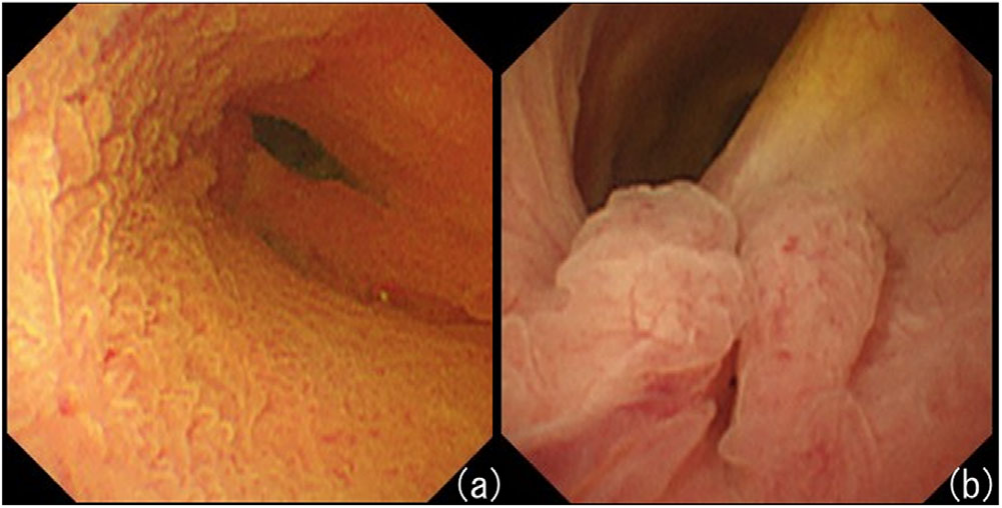
Peroral cholangioscopy findings. (a) Peroral cholangioscopy showing granular lesion in the common bile duct. (b) Nodular lesion found at the confluence of the cystic duct.

An elevated lesion was found in the bile duct at the time of stone removal, and the patient was observed again using POCS one week later. The lesion in the bile duct was a granular mucosal elevation, which was thought to be an inflammatory change (Figure 1a). However, a nodular lesion was found at the confluence of the cystic duct (Figure 1b). Therefore, the patient was hospitalized two months later for reevaluation of the bile duct lesion.

**FIGURE 2:**
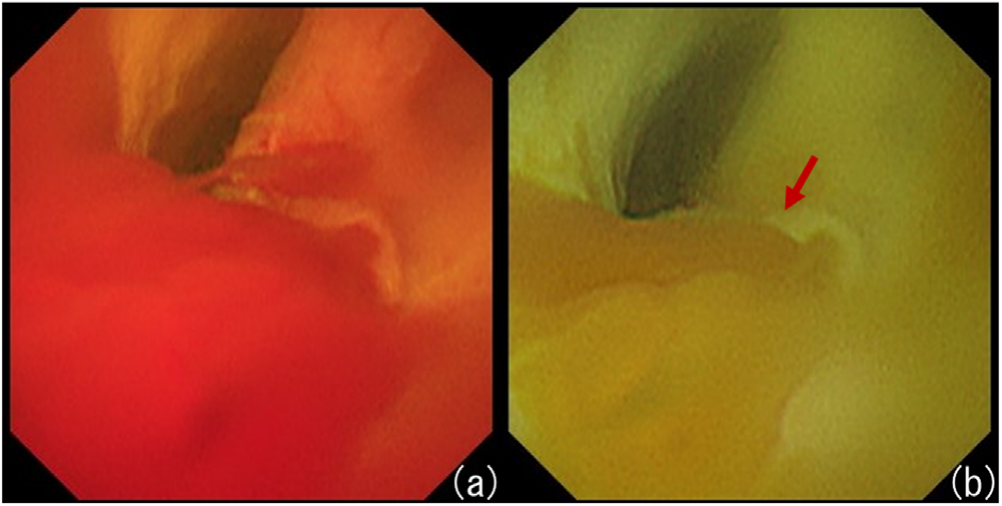
Comparison of the appearances of the biopsy site during white light imaging and red dichromatic imaging. (a) After the biopsy of the nodal lesion, bleeding was observed from the biopsy site. The bleeding point was immediately no longer detectable on white light imaging. (b) After switching to red dichromatic imaging 2 and washing the bile duct with saline solution, the bleeding point was observed in darker yellow (red arrow) than the surrounding area, allowing identification of the bleeding point. RDI, red dichromatic imaging.

Although the granular mucosa of the common bile duct and nodular lesion at the confluence of the cystic duct were reduced, a biopsy of the nodular lesion was performed using SpyBite forceps (Boston Scientific, Marlborough, MA, USA) for pathological diagnosis. Bleeding occurred after the biopsy, but it was difficult to confirm the bleeding point by observation with white‐light imaging because of the pooling of blood (Figure 2a). After switching to RDI 2 and washing the bile duct with saline solution, the bleeding point was clearly observed in darker yellow than the surrounding blood, allowing identification of the bleeding point (Figure 2b and Video S1). Hemorrhage after biopsy stopped spontaneously and did not require any additional hemostatic treatment; furthermore, post‐POCS blood tests showed no decrease in hemoglobin. Pathological findings revealed reactive changes, and cholangiocarcinoma was ruled out.

## DISCUSSION

POCS was introduced in the 1970s in two different methods. The earliest platform was designed as a “mother–baby” system, in which two endoscopists operated a “baby” cholangioscope and a “mother” duodenoscope.^1^ Urakami et al. reported the first direct cholangioscopy using an anterior view endoscope.^2^ Since then, POCS has become widely used for both diagnosis and treatment, including the treatment of difficult biliary stones, evaluation and targeted biopsy for indeterminate biliary strictures and intraductal tumors, and biliary tumor ablation.^3^ It has been reported that observation and hemostasis with peroral direct cholangioscopy are possible for repeated biliary bleeding.^4^

RDI uses three wavelengths; 540, 600, and 630 nm. Hemoglobin strongly absorbs green light with a wavelength of 540 nm; therefore, the reflected light is mostly attenuated independent of the blood concentration. Hemoglobin also strongly absorbs amber light with a wavelength of 600 nm; therefore, the reflected light is greatly attenuated at bleeding points with high hemoglobin concentration compared to that at the surrounding area where hemoglobin concentration is low. Red light with a wavelength of 630 nm is weakly absorbed by hemoglobin, and the reflected light is barely attenuated, regardless of the hemoglobin concentration. Using the differences in hemoglobin absorption between 600 nm and 630 nm, RDI clarifies the contrast between the darker yellow color of the bleeding spot and the yellow color of the surrounding diluted blood.

This property of RDI was first reported in duodenal ulcer bleeding.^5^ Since then, there have been several reports on the use of RDI to treat a variety of bleeding conditions, and its usefulness in acute gastrointestinal bleeding has been reported.^6^ Recently, there have also been reports of RDI being useful in post‐endoscopic sphincterotomy bleeding.^7^

Koiwai et al. reported that RDI 3 in POCS allows clear visibility in the presence of bile.^8^ Matsumoto et al. further reported the usefulness of RDI 3 in POCS,^9^ while Tanisaka et al. reported the usefulness of texture and color enhancement imaging and RDI in POCS.^10^ However, except for these reports, the usefulness of RDI for POCS has not yet been reported.

This report is the first to demonstrate that RDI is effective in identifying the bleeding point of a biliary hemorrhage, as observed in POCS. In this case, within the remaining gastrointestinal tract, the bleeding point was visible as a dark yellow region compared to the surrounding blood in the biliary tract. In the future, RDI may be useful for hemostasis under POCS.

## CONFLICT OF INTEREST STATEMENT

None.

## Supporting information


**Video 1**: Peroral cholangioscopy and the proceedings. Peroral cholangioscopy showed nodular lesion at the confluence of the cystic duct. A biopsy was performed for pathological diagnosis. Bleeding occurred after the biopsy, but it was difficult to confirm the bleeding point using white‐light imaging. After switching to red dichromatic imaging 2 and washing the bile duct with saline solution, the bleeding point was observed to be in darker yellow than the surrounding area. RDI, red dichromatic imaging.

## Data Availability

Data sharing is not applicable to this article as no datasets were generated or analyzed during the current study.
